# On Levodopa Interactions with Brain Disease Amyloidogenic Proteins at the Nanoscale

**DOI:** 10.1021/acsomega.5c01028

**Published:** 2025-04-02

**Authors:** Talia Bergaglio, Nico Kummer, Shayon Bhattacharya, Damien Thompson, Silvia Campioni, Peter Niraj Nirmalraj

**Affiliations:** † Transport at Nanoscale Interfaces Laboratory, Swiss Federal Laboratories for Materials Science and Technology, CH-8600 Dübendorf, Switzerland; ‡ Graduate School for Cellular and Biomedical Sciences, University of Bern, CH-3012 Bern , Switzerland; § Department of Physics, Bernal Institute, University of Limerick, V94T9PX Limerick , Ireland; ∥ Functional Materials Laboratory, Swiss Federal Laboratories for Materials Science and Technology, CH-8600 Dübendorf, Switzerland

## Abstract

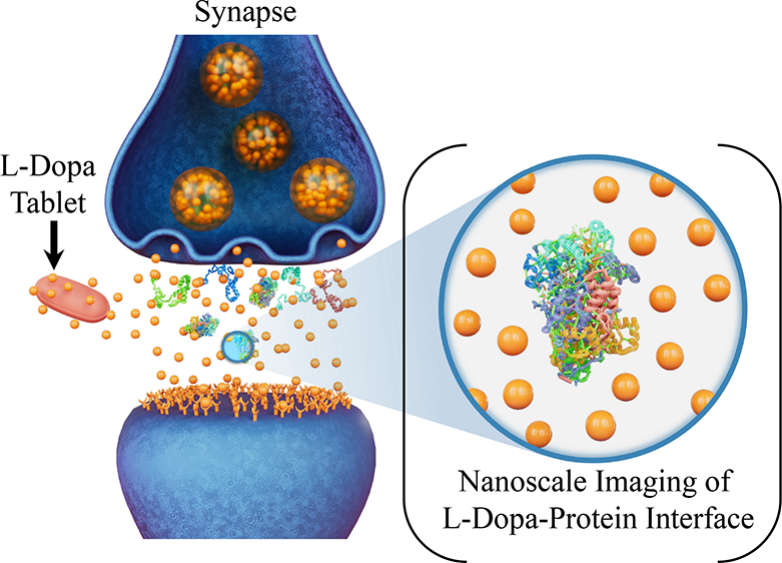

The cerebral accumulation of α-synuclein (α-Syn) and amyloid β-1–42 (Aβ-42) proteins is known to play a key role in the pathology of Parkinson’s disease (PD). Currently, levodopa (L-dopa) is the first-line dopamine replacement therapy for treating bradykinetic symptoms (i.e., difficulty initiating physical movements), which become visible in PD patients. Using atomic force microscopy, we evidence at nanometer length scales the differential effects of L-dopa on the morphology of α-Syn and Aβ-42 protein fibrils. L-dopa treatment was observed to reduce the length and diameter of both types of protein fibrils, with a stark reduction mainly observed for Aβ-42 fibrils in physiological buffer solution and human cerebrospinal fluid. The insights gained on Aβ-42 fibril disassembly from the label-free nanoscale imaging experiments are substantiated by using atomic-scale molecular dynamics simulations. Our results indicate L-dopa-driven reversal of amyloidogenic protein aggregation, which might provide leads for designing chemical effector-mediated disassembly of insoluble protein aggregates.

## Introduction

Alzheimer’s and Parkinson’s disease are characterized by the progressive accumulation of protein aggregates in brain synapses.^1^ Yet, there is a significant difference in the chemical structure of the key proteins implicated in each neurodegenerative disorder. In Parkinson’s disease (PD), aggregation of the misfolded α-synuclein (α-Syn) protein,^2−4^ from its native soluble disordered state to β-sheet-rich insoluble fibril structures, drives the formation of toxic intracellular aggregates known as Lewy bodies and Lewy neurites. This leads to the degeneration of dopaminergic neurons within the substantia nigra, switching off synthesis of the neurotransmitter dopamine in the midbrain basal ganglia structure.^5−7^ Conversely, in Alzheimer’s disease (AD), the aggregation of amyloid β peptide (chiefly, Aβ-42)^8,9^ and tau protein^10^ in the brain tissue results in the formation of amyloid plaques and neurofibrillary tangles, respectively, triggering neurocognitive impairments.^11^ There is emerging evidence that individuals with PD frequently exhibit nonmotor symptoms typical of AD patients, with α-Syn and Aβ accumulations codetected in the brain of PD and AD patients.^12,13^ Importantly, pathogenic processes triggering the onset of AD and PD typically occur several decades before the emergence of visible signs of memory and cognitive deficits,^14^ indicating that the gradual changes in α-Syn and Aβ protein structure from monomers, oligomers, and protofibrils to fibrils progressively disrupt neuronal function and connectivity.^15^ These pathological protein aggregates are present in both the blood^16^ and cerebrospinal fluid (CSF)^17^ of PD and AD patients, which provides an opportunity to monitor disease progression before the onset of clinical symptoms through the development of fluid biomarkers aimed at the detection and quantification of α-Syn and Aβ-42 proteins.^16^

**Figure 1:**
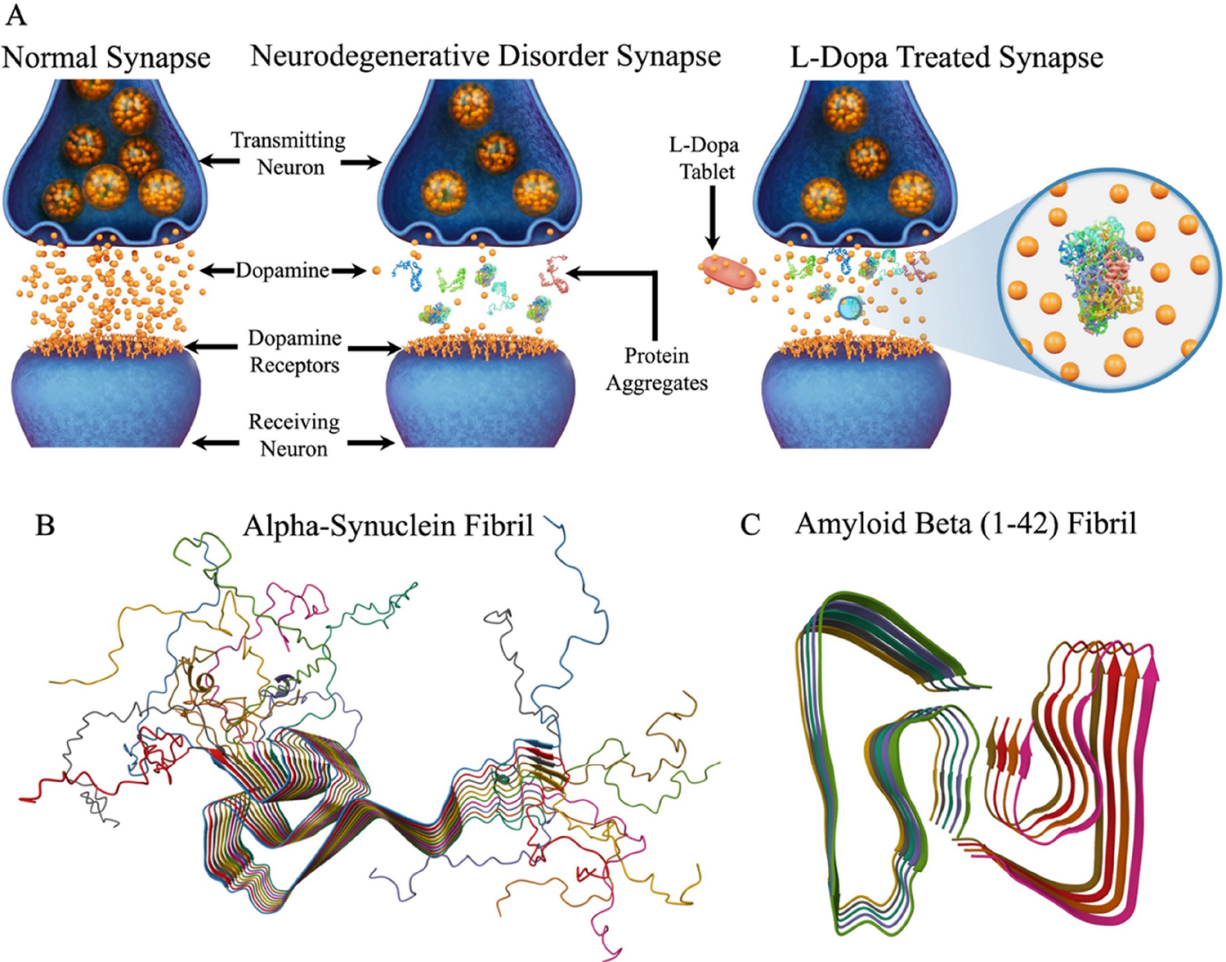
Study rationale and pathological protein structure. (A) Schematic detailing the differences between normal synapse, neurocognitive disorder synapse, and levodopa-treated synapse. The objects shown are not to scale. Understanding the impact of levodopa on protein aggregates such as alpha-synuclein (B, protein data bank identifier: 2NOA) and amyloid beta (1–42) fibril (C, protein data bank identifier: 5OQV) implicated in neurodegenerative disorders such as Parkinson’s and Alzheimer’s disease using nanoscale imaging is the focus of the present study.

Levodopa (L-dopa) is a first-line drug in the pharmacological management of PD. As a precursor to dopamine, L-dopa replenishes dopamine levels in the brain’s depleted regions, providing effective relief from motor symptoms in PD patients.^19^ While the distribution and degree of α-Syn buildup in different regions of the brain of individuals with PD are well-documented, the effect of long-term dopamine replacement therapy on α-Syn aggregation remains unknown.^20^ Importantly, prolonged L-dopa therapy can be associated with motor fluctuations, dyskinesia, and a consequent reduction in the effectiveness of a given L-dopa dose.^21^ The generation of free radicals, as well as L-dopa-induced toxicity to dopaminergic neurons, may, in turn, accelerate the neurodegenerative processes underlying PD.^22^ Although L-dopa cannot arrest or slow down the advancement of PD nor reverse the course of the disease, in vitro studies have demonstrated that L-dopa can effectively hinder the formation of α-Syn fibrils and promote the disassembly of pre-existing fibrils.^23,24^ The effect of L-dopa on synapses is schematically shown in Figure 1A, comparing healthy, diseased, and L-dopa-treated neuronal interfaces. While L-dopa is primarily used in the management of PD, the effects of the dopamine precursor on Aβ pathology have also been investigated due to the synergy of PD and AD pathology.^25^ Specifically, in vitro studies using Thioflavin T (ThT) fluorescence have shown that L-dopa may alter the morphology of Aβ aggregates by inhibiting Aβ fibrillation.^23,24^ While the investigation into the therapeutic efficacy of L-dopa on cognitive decline in dementia remains ongoing,^26,27^ the substantial involvement of dopamine in learning and memory consolidation has sparked considerable interest in its potential application beyond PD,^28,29^ including mitigation of AD-related pathology by L-dopa targeting of Aβ aggregation.^30^ Hence, understanding the complex mechanisms governing α-Syn and Aβ aggregation and the mode of action of dopamine replacement therapies is essential for effective therapeutic interventions designed to halt PD progression and, specifically, PD-related dementia. Although previous studies using Thioflavin T (ThT), kinetics binding assays have suggested a significant reduction in α-Syn and Aβ aggregation in the presence of L-dopa,^23,24^ nanoscale characterization techniques, such as atomic force microscopy (AFM), can provide high-resolution visualization and quantitative measurement of the mechanical properties, aggregation patterns, and morphological changes induced by L-dopa.^23,31,32^ Molecular insights into protein–drug interactions are crucial for predicting the clinical outcomes of L-dopa treatment in PD and AD patients. Previously, we resolved and quantified the size, shape, and morphology of diverse protein aggregates formed along the primary aggregation pathway of wild-type α-Syn and Aβ-40 and Aβ-42 in vitro using AFM.^31,33^

In the present work, we extend the application of AFM to investigate the effects of L-dopa on α-Syn and Aβ-42 fibrils at nanometer length scales. The atomically resolved structures of α-Syn and Aβ-42 fibrils used as models in our previous works^31,33^ are shown in Figure 1B,C, respectively. When treated with 100 μM L-dopa, a reduction in the length and diameter of fibrils was observed for α-Syn and Aβ-42 (see the Materials and Methods Section for protein solution preparation). Control studies conducted directly in human CSF revealed a decrease in fibril length and diameter when compared to untreated Aβ-42-CSF samples, indicative of fibril disassembly through the action of L-dopa confirmed from AFM measurements. Molecular dynamics simulations revealed the formation of a physiosorbed layer of L-dopa on the fibrils. This layer facilitates their destabilization and masking of the aggregation-prone hydrophobic cores of the released low molecular weight oligomers, thus inhibiting their reincorporation into the fibrils.

## Results and Discussion

### Nanoscale Imaging of Untreated and L-dopa-Treated α-Syn Protein Aggregates Prepared in Physiological Buffer Solution

**Figure 2:**
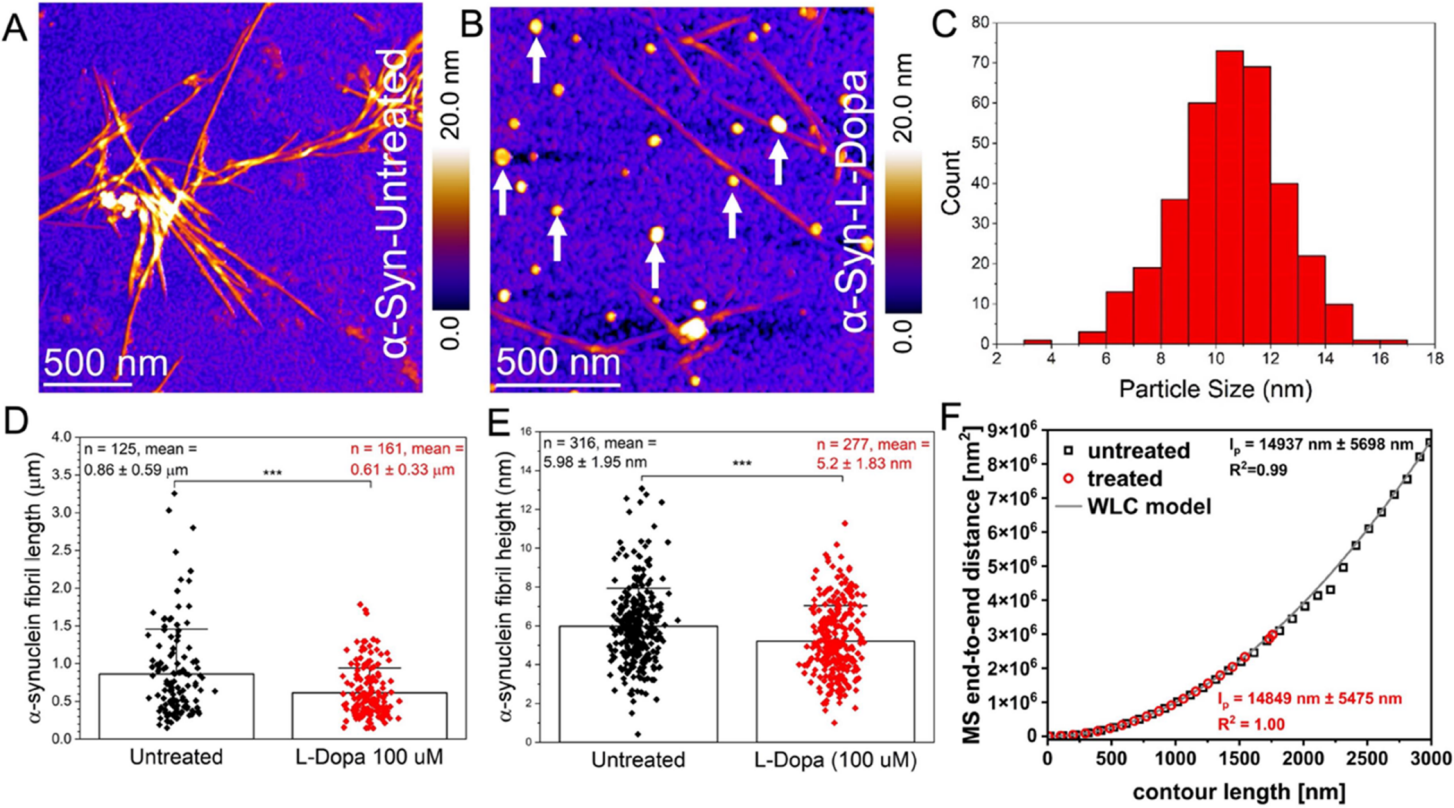
Nanoscale imaging of untreated and L-dopa-treated α-Syn in buffer salt solution. (A) AFM height map of untreated α-Syn protein fibrillar aggregates deposited on a gold surface. (B) AFM height image of α-Syn protein monomers incubated with 100 μM L-dopa and deposited on a gold surface. (C) Size distribution of spherical particles of 10.53 ± 1.96 nm, based on AFM height data. (D, E) Plot of the mean α-Syn fibril length and height values obtained in untreated samples (coded in black) and samples incubated with L-dopa (coded in red). Error bars indicate the standard deviation from the mean. (F) Persistence length of untreated α-Syn fibrils (hollow sphere trace) and L-dopa-treated (red trace) α-Syn fibrils. The worm-like chain model (WLC) is plotted as a dark gray line.

We first performed AFM characterization of α-Syn fibrils that were incubated with and without 100 μM L-dopa for 6 days under mechanical agitation at 37 °C. Figure 2a shows an AFM height image recorded after depositing the untreated α-Syn solution, showing aggregated α-Syn fibrils. The AFM height image for the L-dopa-treated sample (Figure 2B) shows the presence of α-Syn fibrils and L-dopa particles (white arrows, visible on the fibrils and throughout the sample). To assess if the detected spherical particles in Figure 2B could represent L-dopa particles, we calculated the mean size of all of the spherical particles resolved in the AFM images where α-Syn proteins were incubated together with L-dopa. Based on single particle size analysis, we calculated a mean L-dopa particle size of 10.5 ± 1.95 nm (Figure 2C). Such spherical particle sizes were not detected when α-Syn proteins were incubated in the absence of L-dopa, suggesting that spherical particles are L-dopa molecules present as aggregates. To quantify the effect of L-dopa treatment on α-Syn aggregation, we extracted the height and length distribution, as well as the persistence length measurement, of α-Syn fibrils incubated without and with 100 μM L-dopa. A significant decrease in the fibril length (Figure 2D) and height (Figure 2E) was observed for the α-Syn fibrils incubated with L-dopa. A two-sample *t* test revealed a significant reduction in fibril length (Figure 2D) when incubated with 100 μM L-dopa (610 ± 330 nm) compared to that of the untreated fibrils (860 ± 590 nm). Similarly, the mean height (Figure 2E) of the individual fibrils was also reduced upon treatment with L-dopa (5.20 ± 1.8 nm) compared to the control condition (5.96 ± 1.95 nm). Additionally, we assessed the nanomechanical properties of the α-Syn fibrils with and without L-dopa treatment. Figure 2F shows the mean square end-to-end distance as a function of the contour length for untreated fibrils (hollow sphere trace) and L-dopa-treated fibrils (red). The worm-like chain model (WLC) is plotted as a dark gray line. The calculated persistence length suggested negligible differences in mechanical properties for untreated α-Syn fibrils with average persistence lengths of 14.94 ± 5.70 and 14.85 ± 5.48 μm for L-dopa-treated α-Syn fibrils. Previously, we studied the effect of ibuprofen on blood and observed a concentration and time-dependent effect on the morphology of red blood cells (RBCs), characterized by the formation of spicules on the RBC membrane and the transition from normocytes into echinocytes.^34^ To evaluate the effect of L-dopa on blood, we conducted similar measurements, as detailed in Figure S1; however, we did not detect any L-dopa-induced structural changes in RBC morphology. Our findings indicate that, while L-dopa plays a role in the disassembly of α-Syn protein aggregation, there were no corresponding structural alterations in RBC morphology, suggesting that L-dopa likely has minimal deleterious hematological effects upon interaction with blood.

### Characterization of Untreated and L-dopa-Treated Aβ-42 Protein Aggregates in Physiological Buffer Solution

**Figure 3:**
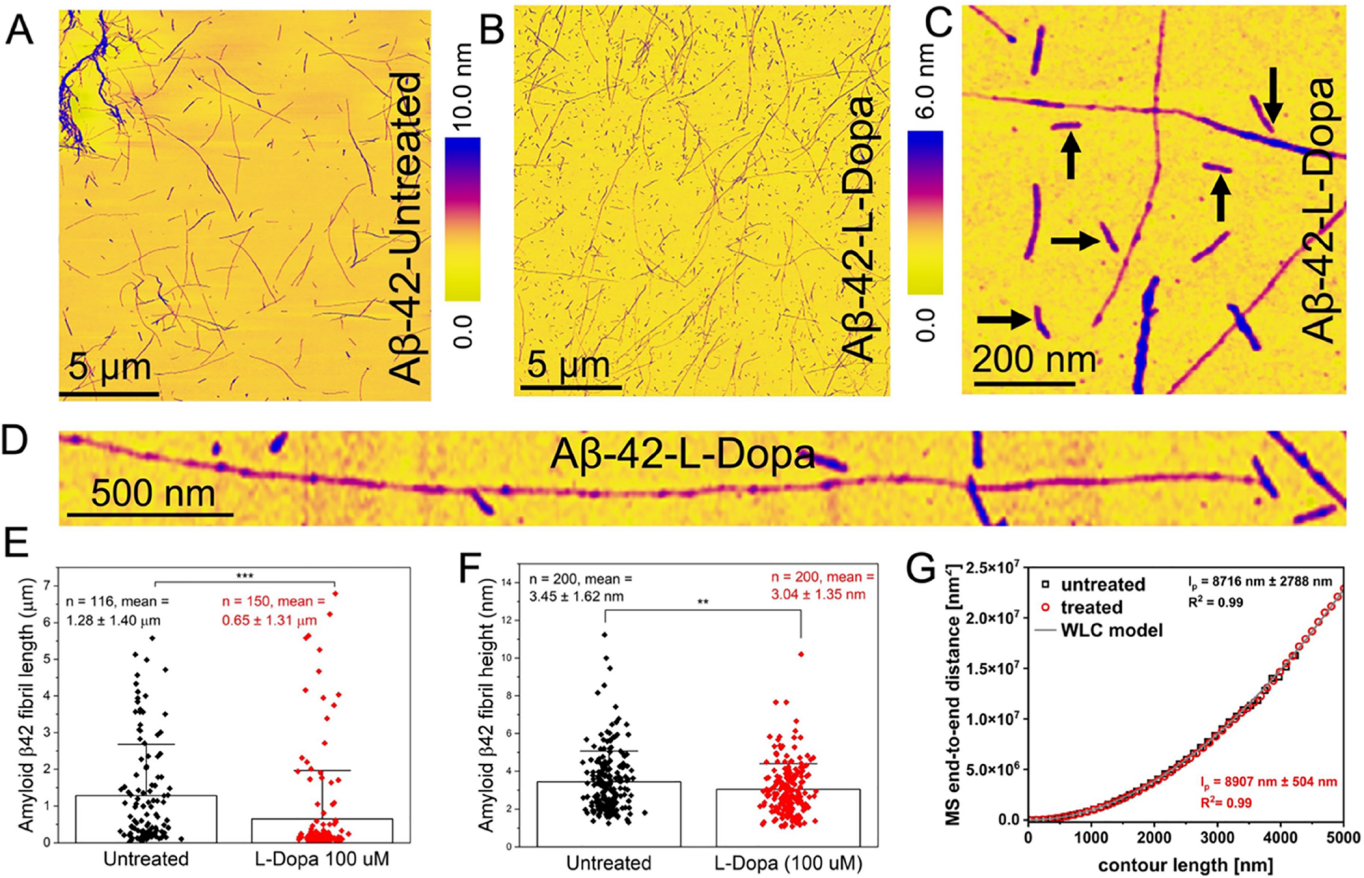
Characterization of untreated and L-dopa-treated Aβ-42 in physiological buffer. (A) Large-area AFM image showing untreated Aβ-42 fibrils. (B) AFM image showing L-dopa-treated Aβ-42 fibrils of diverse lengths formed after incubating Aβ-42 peptides with L-dopa. (C) AFM image of L-dopa-treated Aβ-42 proteins confirming the presence of short fibrils (indicated by black arrows). (D) AFM image of a single Aβ-42 fibril treated with L-dopa. (E) Distribution of fibril length for untreated Aβ-42 (black color-coded) and L-dopa-treated Aβ-42 fibrils (red color-coded). (F) Distribution of fibril height for untreated Aβ-42 (black color-coded) and L-dopa-treated Aβ-42 fibrils (red color-coded). (G) Combined plot of MS end-to-end distance versus contour length for untreated Aβ-42 fibrils (hollow square trace) and L-dopa-treated Aβ-42 fibrils (red sphere trace).

Next, we characterized using AFM the Aβ-42 protein aggregates under identical peptide concentration as the α-Syn experiments (see the Materials and Methods Section for details on Aβ-42 peptide solution preparation). Note: The incubation temperature was kept at 37 °C for both α-Syn and Aβ-42 peptide solution preparation, but the incubation time was different (6 days for α-Syn and 24 h for Aβ-42). It is known from previous in vitro studies that Aβ-42 tends to aggregate faster even along the primary pathway,^31^ evidenced by early onset fibril formation when compared to Aβ-40 and α-Syn proteins. We recently showed that Aβ-42 proteins tend to generate oligomers on the surface of primary fibrils through an accelerated secondary nucleation pathway.^35^ Based on these previous studies, we implemented a shorter incubation protocol for Aβ-42 compared to α-Syn proteins, which was sufficient to generate the necessary mature fibrils. The AFM height map of untreated Aβ-42 (Figure 3A) confirms the predominant presence of mature Aβ-42 fibrils. The presence of fibrils and the absence of smaller oligomeric particles on the solid surface suggest that the 24 h incubation period results in the saturation phase of Aβ-42 assembly. The L-dopa-treated Aβ-42 sample (Figure 3B) reveals shorter fibril fragments (indicated by black arrows in Figure 3C), which were not detected for the untreated Aβ-42 proteins, together with an overall reduction of the height of the fibrils. Figure 3D is a high-resolution AFM topograph of an individual L-dopa-treated Aβ-42 fibril. The diameter, when measured at multiple points along the elongated fibril (no nodular morphology typical of protofibrils), was ∼2 nm.

Based on AFM measurements on several such individual fibrils, we calculated a mean fibril length of (1.28 ± 1.4 μm) for untreated (*n* = 116, black plot Figure 3E) and (0.65 ± 1.31 μm) for L-dopa-treated Aβ-42 fibrils (*n* = 150, red plot Figure 3E). Likewise, and in common with the trends observed for α-Syn, the AFM data confirmed an overall reduction in the fibril diameter (Figure 3F) but no measurable differences in persistence length (Figure 3G) after treatment with L-dopa. Based on the nanoscale imaging experiments conducted on the aggregated forms of α-Syn and Aβ-42 proteins in the presence and absence of L-dopa, we observed the most noticeable morphological changes (reduction in fibril length and diameter) to be associated with L-dopa-treated Aβ-42 species. All measurements detailed above were conducted on synthetically prepared proteins in physiological buffer salt solutions, which are much simpler in composition (mainly water, sodium chloride, and phosphate) compared to body fluids, such as CSF (water, proteins, ions, and other organic electrolytes).

### Direct Imaging of Untreated and L-dopa-Treated Aβ-42 in Human CSF

**Figure 4:**
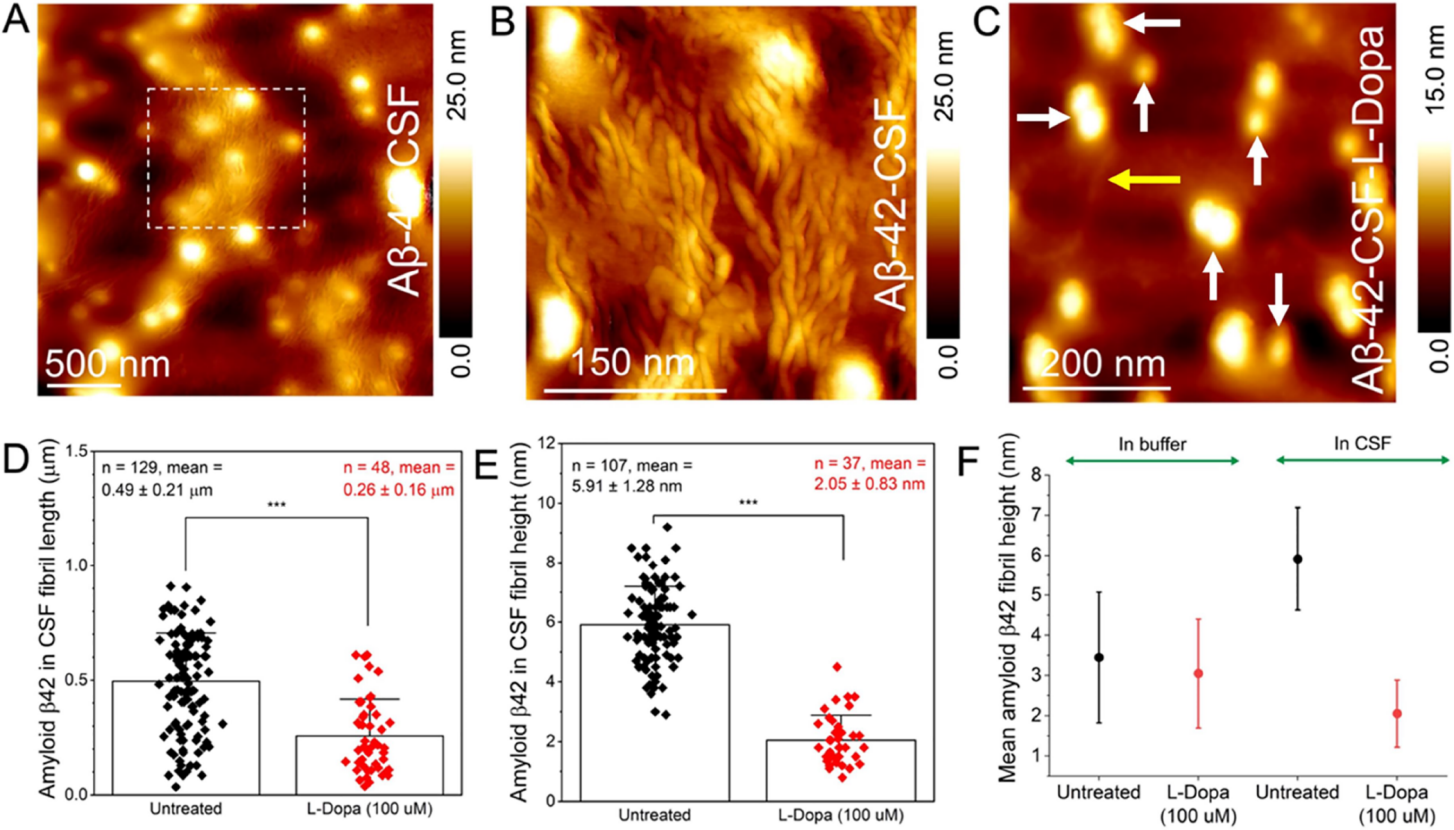
Direct imaging of untreated and L-dopa-treated Aβ-42 in human CSF. (A) AFM image revealing the presence of both fibrillar and spherical Aβ-42 aggregates in CSF. (B) High-resolution AFM image of untreated Aβ-42 fibrils resolved within the white dash box in panel A. (C) Large-area AFM image of L-dopa (100 μM)-treated Aβ-42 aggregates showing the presence of mostly spherical (indicated by white arrows) and lower prevalence of fibrils (indicated by yellow arrow). (D) Distribution of fibril length for untreated Aβ-42 (black color-coded) and L-dopa-treated Aβ-42 fibrils (red color-coded) in CSF. (E) Distribution of fibril height for untreated Aβ-42 (black color-coded) and L-dopa-treated Aβ-42 fibrils (red color-coded) in CSF. (F) Mean Aβ-42 fibril height measured in buffer solution and CSF, without (colored black, mean_buffer_: 3.45 ± 1.62 nm; mean_CSF_: 5.91 ± 1.28 nm) and with incubation with100 μM L-dopa (colored red, mean_buffer_: 3.05 ± 1.35 nm; mean_CSF_: 2.05 ± 0.83 nm).

Although information obtained from morphological studies on pathological proteins in buffer salt solutions can provide some insights into protein aggregation mechanisms, it is important to directly study such processes in CSF, as it is a more biologically relevant environment for both therapy and diagnostics. To test the effect of L-dopa on protein assembly in CSF, we purchased commercially available samples of Aβ-42 proteins enriched in a healthy human CSF from Sigma-Aldrich (see the Materials and Methods Section for details on CSF sample preparation). Five μL of Aβ-42 (5 μL) in the CSF sample was drop-casted on the gold substrate and imaged using AFM, followed by air-drying the sample for 5 h under standard laboratory conditions. Figure 4A shows an AFM image showing the presence of both large spherical particles and dense fibrils, which we classify as Aβ-42 protein aggregates. The fibrils appear to be closely packed in CSF, as observed in the zoomed-in image (Figure 4B), which contrasts with the isolated nature of the Aβ-42 fibrils deposited from buffer salt solution on the gold substrate. Inspecting the Aβ-42 CSF sample incubated with L-dopa (concentration: 100 μM) for 6 days at 37 °C under mechanical agitation and deposition on a gold surface revealed a reduction in the prevalence, length, and height of the Aβ-42 fibrils. Figure 4C is a representative AFM image of L-dopa-treated Aβ-42 protein aggregates in CSF. The spherical particles in CSF were still present after L-dopa incubation (indicated by white arrows). However, densely packed Aβ-42 fibrils previously resolved in untreated CSF samples (Figure 4A,B) were no longer prevalently detected in L-dopa-treated CSF samples. A small population of fibrils was still observed, but the fibrils were of reduced diameter, as indicated by the yellow arrow in Figure 4C. The distribution in length and height of untreated Aβ-42 fibrils (black plots) in CSF and L-dopa-treated Aβ-42 fibrils in CSF (red plots) is shown in Figure 4D,E, respectively. The combined height distribution of untreated (black plots) and L-dopa-treated (red plots) Aβ-42 protein fibrils in buffer solution and CSF is summarized in Figure 4F. Taken together, the AFM measurements on Aβ-42 aggregates in CSF confirmed that L-dopa also disassembles Aβ-42 fibrils, evidenced through the measured reduction in fibril length and height after L-dopa treatment.

The AFM study has limitations. Although we have shown that it is possible to resolve the effects of L-dopa on protein aggregates with nanometer-scale spatial resolution, our current experimental setup lacks the time resolution to capture the disassembly in real time, which could provide deeper insights into the disassembly mechanism. To address this unmet need and to quantify the interfacial interactions of L-dopa with the deposited protein fibrils, we performed atomic-scale molecular dynamics simulations on experimentally inaccessible time scales.

### Studying Effect of L-dopa on Aβ-42 Protein Fibril Folds Using Molecular Dynamics Simulations

**Figure 5:**
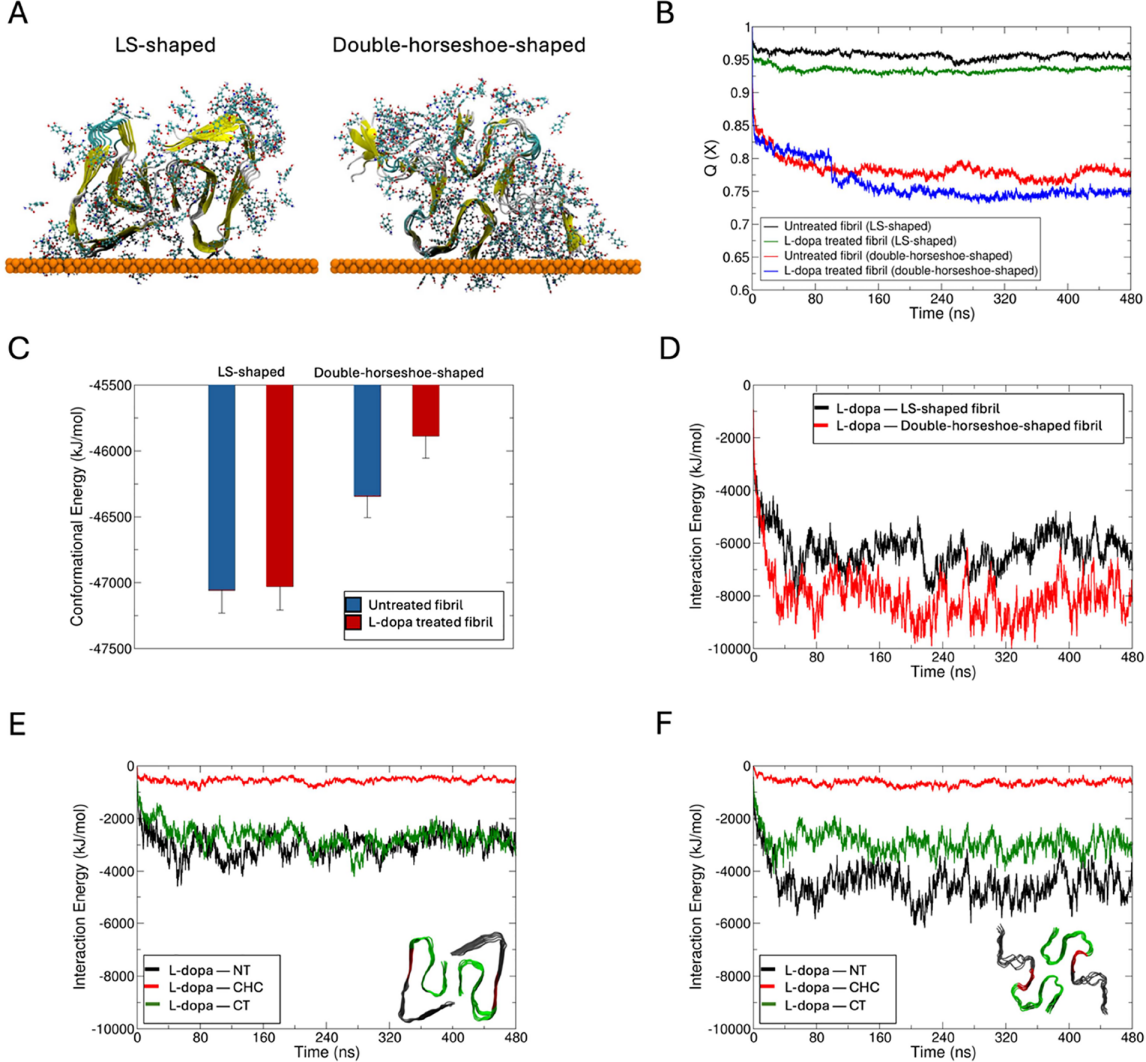
Impact of L-dopa on Aβ-42 protein fibril folds from molecular dynamics simulations. (A) Final conformation of Aβ-42 fibril in their LS-shaped fold (PDB code 5OQV(36)) and double-horseshoe-shaped fold (right, PDB code 2NAO(37)) in the presence of L-dopa molecules at the gold–water interface following 480 ns of dynamics. L-dopa molecules within 5 Å of the fibril are shown in ball and stick representation. The protein fibril is shown in its secondary structure representation, and the gold surface is shown as an atomic sphere. Comparison of the time evolution of (B) fraction of native contacts (*Q*(*X*)) and (C) conformational energies between untreated and L-dopa-treated fibril folds. Time evolution of interaction energies between L-dopa and (D) the two fibril folds, and between L-dopa and the N-terminus (NT, black), central hydrophobic cluster (CHC, red) and C-terminus (CT, green) of (E) LS-shaped, and (F) double-horseshoe-shaped fibril folds. The two fibril folds with different colored regions are shown as insets.

For the MD studies, we chose to focus on the effects of L-dopa on Aβ-42 fibrils, as we evidenced from AFM studies that the disassembly was more pronounced for this system compared to L-dopa-treated α-Syn fibrils. We sample multiple Aβ-42 fibril fold morphologies by creating starting structures in the LS-shaped fold solved by cryo-EM (PDB code 5OQV(36)) and the double-horseshoe-shaped structure solved by solution NMR (PDB code 2NAO(37)). The protein assembly was placed at the gold substrate in a large water box for simulations without and with 1.3 mM L-dopa (see Materials and Methods Section), and the L-dopa interacted freely with the protein during 0.1 μs of free dynamics (Figures 5A and S2C–F). The models reveal a greater loss in native contacts (Q(X))^38^ (Figure 5B) with the partial unfolding of β-sheet to random coil (Figure S2E–H) for Aβ-42 treated with L-dopa compared to untreated protofibrils. To account for the fibril thermodynamic stabilities without and with L-dopa, we computed the protein conformational energies by the Generalized Born using Molecular Volume (GBMV) Solvation Energy method implemented in the CHARMM (v40b2) program.^39^ While the thermodynamic stability of the L-dopa-treated LS-shaped Aβ-42 fibril fold is at par with the untreated fold (Figure 5C), the AD-relevant double-horseshoe-shaped fibril fold showed different thermodynamic stability^40^ and is significantly destabilized when treated with L-dopa (Figure 5C). This NMR-solved double-horseshoe cross-β-fibril polymorph was recognized by the antibodies that typically bind to intracellular deposits and senile plaques in the brains of AD patients.

During the MD simulations, the L-dopa molecules spontaneously formed an ordered cloak around the fibrils driven by Coulombic L-dopa–fibril pairwise interactions (Figures 5D, S2G,H). The L-dopa–fibril interactions are majorly contributed by the N-terminus (NT, residues 1–16) of the double-horseshoe-shaped fold, followed by the C-terminus (CT, residues 22–42) and the central hydrophobic cluster (CHC, the hydrophobic “hotspots” of aggregation, residues 17–21) (Figure 5F). By contrast, the L-dopa–NT interactions are not significantly greater than the L-dopa—CT interactions in the LS-fold due to a more structured and less exposed NT than in the double-horseshoe fold (Figure 5E). The remaining significant L-dopa–CHC interactions suggest that the reassembly of L-dopa-treated etched fibrils may be slowed by blocking the CHC hotspots of aggregation by L-dopa in the released low-molecular-weight species. Overall, our modeling data strongly support the finding from AFM maps that the interaction of Aβ-42 fibril with L-dopa leads to destabilization and disassembly of fibrils with high affinity, and the destabilizing effect of L-dopa molecules around the fibril and favorable L-dopa–CHC contacts may also screen these aggregation sites and prevent reintegration of released oligomers. While our MD simulations demonstrate that L-dopa forms a surface-bound layer that destabilizes Aβ-42 fibrils, the in vivo environment presents additional complexities. Specifically, dopamine and its oxidation products, such as dopamine quinones, can engage in redox cycling, covalent modifications, and additional interactions that may further modulate the fibril aggregation dynamics. These oxidative species could lead to alternative fibril destabilization pathways or contribute to fibril stabilization under certain conditions. Future studies incorporating oxidized dopamine species into computational models or experimental validation through biochemical assays are necessary to fully understand these effects.

## Conclusions

In summary, we have evidence of the disassembly of fibrillar forms of Aβ-42 and α-Syn proteins at the nanoscale upon treatment with L-dopa. The differences in the size and shape of the individual L-dopa-treated protein fibrils are recorded in a label-free manner by using AFM. In particular, the effect of L-dopa was observed to have a more distinct effect on Aβ-42 fibrils in both physiological solution and CSF. Molecular dynamics simulations quantified the interfacial interactions between L-dopa and Aβ-42 fibrils. The disassembly of fibrillar structures observed upon the L-dopa interaction, particularly with Aβ-42 fibrils, indicates that L-dopa disrupts mature fibrils and may promote the formation of smaller oligomeric species. No significant changes in the persistence length of Aβ-42 and α-Syn fibrils before and after L-dopa treatment suggest that the smaller fibril fragments observed from the AFM images could still contain the β-sheet-rich core structure and hence are prone to further aggregation. While the disassembly of fibrillar proteins by L-dopa could represent a therapeutic strategy to halt or reverse disease progression, there is a significant concern that intermediates formed during fibril disassembly, or products of incomplete fibrillation, may exhibit neurotoxic properties.^41^ Thus, in addition to AFM measurements, future studies based on ultrasensitive functional assays can be employed to assess the concentration-dependent toxicity of these oligomers, even in human CSF, thereby leading to the identification of the highly cytotoxic species. The MD simulations show evidence for the destabilizing effect of L-dopa with key Aβ-42 aggregation sites, potentially also inhibiting the reaggregation of fibril fragments, thereby favoring the accumulation of likely toxic oligomeric species. This mechanism could explain the known adverse side effects of long-term L-dopa treatment in PD, including irreversible motor dysfunction, such as dyskinesia.^42^ Altered protein aggregation and the formation of toxic oligomeric species induced by L-dopa may exacerbate neuronal damage and contribute to the observed motor complications, highlighting the delicate balance between L-dopa therapeutic effects and the potential for exacerbating neurodegeneration.^23,43^ Our findings emphasize the complexity of L-dopa interactions with pathological protein aggregates and highlight the need for optimizing treatment strategies that mitigate long-term side effects and resist pathogenic aggregation by stabilizing nontoxic conformational states, thereby reducing aggregation-prone interactions.

## Materials and Methods

### Preparation of Wild-Type α-Synuclein, Aβ-42, and L-dopa Solutions

Wild-type human α-Syn was obtained following the procedures outlined by Campioni et al.^44^ to prepare the α-Syn solutions. The lyophilized protein (∼30 mg/mL) was first dissolved in 700 μL of PBS buffer (VWR). The pH was then adjusted to 7.4 by using 1 M sodium hydroxide (NaOH). To filter the solution, the filter membrane of a 100 kDa NMWL centrifugal filter (Amicon Ultra-4 Centrifugal Filter Unit, Merk Millipore) was first hydrated with 4 mL of PBS buffer and centrifuged for 5 min at 3200*g* three times. Next, the α-Syn solution (∼700 μL) was added to the centrifugal filter and centrifuged for 20 min at 3200*g* to filter out any large α-Syn particles that had not fully dissolved. Lastly, to extract any remaining α-Syn from the bottom of the filter, 100 μL of PBS buffer was added to the filter and mixed, followed by centrifugation for 5 min at 3200*g*. A spectrophotometer (Implen Nanophotometer NP80 UV–vis) was used to determine the final concentration of α-Syn (ε_280_ = 5960 M^–1^ cm^–1^). The obtained α-Syn stock solution was further diluted with a PBS buffer to reach a final concentration of 300 μM.

The aggregation of Aβ-42 in phosphate buffer salt solution (VWR) was performed according to the previously described protocol.^45^ One entire vial (250 μg) of commercial Aβ-42 peptide (Sigma-Aldrich) was dissolved in 10% ammonium hydroxide (Sigma-Aldrich) at a concentration of 0.5 mg/mL by shaking at 400 rpm for 20 min. Aliquots containing 50 μg of Aβ-42 were transferred into protein-low bind Eppendorf tubes and freeze-dried overnight. For each aggregation experiment, the freeze-dried pellet was dissolved in 100 μL 60 mM NaOH (reaching a concentration of 0.5 mL or ∼110 μM, confirmed by Implen NanoPhotometer at 280 nm with an extinction coefficient of 1490 M^–1^ cm^–1^ and a molecular weight of 4515 g/mol). The aggregation was initiated by diluting the sample with PBS to a final concentration of 5 μM and starting incubation at 37 °C and 400 rpm shaking in an Eppendorf Thermomixer for 24 h. To investigate the effect of L-dopa on α-Syn and Aβ-42 aggregation dynamics, a stock solution was prepared by dissolving L-dopa (Merk Millipore) in 1 mL of PBS buffer (1.9719 mg/mL). For the samples incubated with L-dopa, 250 μL of L-dopa solution was added to 250 μL of α-Syn (300 μM), leading to a final concentration of L-dopa of 100 μL. α-Syn, with and without L-dopa, was incubated at 37 °C for 6 days under mechanical agitation at 300 rpm. For Aβ-42 proteins in CSF (1.22 μg/L specified by vendor), we purchased the samples from Sigma-Aldrich, and the as-received samples were aliquoted and stored at −20 °C until further use. The CAS number for this sample is ERMDA482IFCC.

### AFM

AFM measurements on Aβ-42 aggregates in PBS incubated for 24 h were performed using a Bruker Dimension Icon instrument operated in tapping mode and equipped with SCOUT 150 HAR silicon AFM probes (gold reflective backside coating, force constant 18 N/m, resonant frequency: 150 kHz, NuNano). AFM measurements were conducted on air-dried α-Syn incubated without and with 100 μL of L-dopa concentration on day 6 and deposited as a thin film on mica discs. The Aβ-42 solutions (∼10 μL) were deposited on 5 × 5 mm Si wafers for 1 min and then rinsed with 1 mL of ultrapure water; the excess water was dried under a gentle air stream. The raw AFM images were processed and analyzed using the open source software Gwyddion 2.60. 2D leveling and scan line correction were applied, followed by measurements of the fibril height (Nuntreated = 316; NL-dopa = 277) and length (Nuntreated = 125; NL-dopa = 161). The size distribution of L-dopa particles observed in the treated α-Syn sample was calculated for a total of ∼348 particles. Contour and persistence length measurements were performed on several AFM images recorded in different spots on the Si wafer using the MATLAB-based Easyworm software.^46^ Height profiles were extracted by using the Bruker Nanoscope Analysis software. AFM measurements on Aβ-42 aggregates in CSF were performed by using a multimode 8 Bruker instrument equipped with an E-scanner. For the AFM tip, a SCOUT 70 HAR silicon AFM tip was used in tapping mode (gold reflective backside coating, force constant 0.4 N/m, resonant frequency: 70 kHz, NuNano). AFM measurements were conducted on air-dried samples by first depositing 5 μL in separate experiments for both L-dopa-treated and untreated Aβ-42 aggregates in CSF medium on gold thin films, followed by drying in air (∼5 h) and then placing the air-dried CSF samples on gold disks on top of the E-scanner for AFM imaging. Previously, we have reported using liquid-based AFM and standard AFM that the air-drying process leads to a minimal shrinkage of the protein fibrils but does not impact the length of the protein fibrils, and we quantified the shrinkage factor to be 0.8 ± 0.1.^17^

## Supporting Information Available

Preparation of models and molecular dynamics simulations, 3D digital holotomographic analysis of L-dopa effects on blood (PDF)

## References

[ref1] Candelise N.; Scaricamazza S.; Salvatori I.; Ferri A.; Valle C.; Manganelli V.; Garofalo T.; Sorice M.; Misasi R. Protein Aggregation Landscape in Neurodegenerative Diseases Clinical Relevance and Future Applications. Int. J. Mol. Sci. 2021, 22, 6016 10.3390/ijms22116016. 34199513 PMC8199687

[ref2] Bhattacharya S.; Xu L.; Thompson D. Revisiting the earliest signatures of amyloidogenesis: Roadmaps emerging from computational modeling and experiment. WIREs Comput. Mol. Sci. 2018, 8 (4), e1359 10.1002/wcms.1359.

[ref3] Bhattacharya S.; Xu L.; Thompson D. Molecular Simulations Reveal Terminal Group Mediated Stabilization of Helical Conformers in Both Amyloid-beta42 and alpha-Synuclein. ACS Chem. Neurosci. 2019, 10 (6), 2830–2842. 10.1021/acschemneuro.9b00053. 30917651

[ref4] Xu L.; Bhattacharya S.; Thompson D., Predictive Modeling of Neurotoxic α-Synuclein Polymorphs. In Computer Simulations of Aggregation of Proteins and Peptides; Springer US: New York, NY, 2022; 379–399. 10.1007/978-1-0716-1546-1_17 35167083

[ref5] Mehra S.; Sahay S.; Maji S. K. alpha-Synuclein misfolding and aggregation: Implications in Parkinson’s disease pathogenesis. Biochim Biophys Acta Proteins Proteom 2019, 1867 (10), 890–908. 10.1016/j.bbapap.2019.03.001. 30853581

[ref6] Breydo L.; Wu J. W.; Uversky V. N. Alpha-synuclein misfolding and Parkinson’s disease. Biochim. Biophys. Acta 2012, 1822 (2), 261–285. 10.1016/j.bbadis.2011.10.002. 22024360

[ref7] Magalhães P.; Lashuel H. A. Opportunities and challenges of alpha-synuclein as a potential biomarker for Parkinson’s disease and other synucleinopathies. NPJ Parkinsons Dis. 2022, 8 (1), 93 10.1038/s41531-022-00357-0. 35869066 PMC9307631

[ref8] Bhattacharya S.; Xu L.; Thompson D. Long-range Regulation of Partially Folded Amyloidogenic Peptides. Sci. Rep. 2020, 10 (1), 7597 10.1038/s41598-020-64303-x. 32371882 PMC7200734

[ref9] Bhattacharya S.; Xu L.; Thompson D. Characterization of Amyloidogenic Peptide Aggregability in Helical Subspace. Methods Mol. Biol. 2022, 2340, 401–448. 10.1007/978-1-0716-1546-1_18. 35167084

[ref10] Maraba O.; Bhattacharya S.; Conda-Sheridan M.; Thompson D. Modelling peptide self-assembly within a partially disordered tau filament. Nano Express 2022, 3 (4), 044004 10.1088/2632-959X/acb839.

[ref11] Masters C. L.; Bateman R.; Blennow K.; Rowe C. C.; Sperling R. A.; Cummings J. L. Alzheimer’s disease. Nat. Rev. Dis Primers 2015, 1, 15056 10.1038/nrdp.2015.56. 27188934

[ref12] Smith C.; Malek N.; Grosset K.; Cullen B.; Gentleman S.; Grosset D. G. Neuropathology of dementia in patients with Parkinson’s disease: a systematic review of autopsy studies. J. Neurol Neurosurg. Psychiatry 2019, 90 (11), 1234–1243. 10.1136/jnnp-2019-321111. 31444276

[ref13] Han Y.; He Z. Concomitant protein pathogenesis in Parkinson’s disease and perspective mechanisms. Front. Aging Neurosci. 2023, 15, 1189809 10.3389/fnagi.2023.1189809. 37181621 PMC10174460

[ref14] Tolosa E.; Garrido A.; Scholz S. W.; Poewe W. Challenges in the diagnosis of Parkinson’s disease. Lancet Neurol 2021, 20 (5), 385–397. 10.1016/S1474-4422(21)00030-2. 33894193 PMC8185633

[ref15] Tsoi P. S.; Quan M. D.; Ferreon J. C.; Ferreon A. C. M. Aggregation of Disordered Proteins Associated with Neurodegeneration. Int. J. Mol. Sci. 2023, 24 (4), 3380 10.3390/ijms24043380. 36834792 PMC9966039

[ref16] Nirmalraj P. N.; Schneider T.; Felbecker A. Spatial organization of protein aggregates on red blood cells as physical biomarkers of Alzheimer’s disease pathology. Sci. Adv. 2021, 7 (39), eabj2137 10.1126/sciadv.abj2137. 34559561 PMC8462905

[ref17] Nirmalraj P. N.; Schneider T.; Lüder L.; Felbecker A. Protein fibril length in cerebrospinal fluid is increased in Alzheimer’s disease. Commun. Biol. 2023, 6 (1), 251 10.1038/s42003-023-04606-7. 36890343 PMC9995532

[ref19] Yuan H.; Zhang Z. W.; Liang L. W.; Shen Q.; Wang X. D.; Ren S. M.; Ma H. J.; Jiao S. J.; Liu P. Treatment strategies for Parkinson’s disease. Neurosci Bull. 2010, 26 (1), 66–76. 10.1007/s12264-010-0302-z. 20101274 PMC5552548

[ref20] Deffains M.; Canron M. H.; Teil M.; Li Q.; Dehay B.; Bezard E.; Fernagut P. O. L-DOPA regulates α-synuclein accumulation in experimental parkinsonism. Neuropathology and Applied Neurobiology 2021, 47 (4), 532–543. 10.1111/nan.12678. 33275784

[ref21] Yedlapudi D.; Joshi G. S.; Luo D.; Todi S. V.; Dutta A. K. Inhibition of alpha-synuclein aggregation by multifunctional dopamine agonists assessed by a novel in vitro assay and an in vivo Drosophila synucleinopathy model. Sci. Rep. 2016, 6, 38510 10.1038/srep38510. 27917933 PMC5137034

[ref22] Ossig C.; Reichmann H. Treatment strategies in early and advanced Parkinson disease. Neurol Clin 2015, 33 (1), 19–37. 10.1016/j.ncl.2014.09.009. 25432721

[ref23] Li J.; Zhu M.; Manning-Bog A. B.; Di Monte D. A.; Fink A. L. Dopamine and L-dopa disaggregate amyloid fibrils: implications for Parkinson’s and Alzheimer’s disease. FASEB J. 2004, 18 (9), 962–964. 10.1096/fj.03-0770fje. 15059976

[ref24] Conway K. A.; Rochet J. C.; Bieganski R. M.; Lansbury P. T. Kinetic stabilization of the alpha-synuclein protofibril by a dopamine-alpha-synuclein adduct. Science 2001, 294 (5545), 1346–1349. 10.1126/science.1063522. 11701929

[ref25] Walker L.; Attems J. Prevalence of Concomitant Pathologies in Parkinson’s Disease: Implications for Prognosis, Diagnosis, and Insights into Common Pathogenic Mechanisms. J. Parkinsons Dis. 2024, 14 (1), 35–52. 10.3233/JPD-230154. 38143370 PMC10836576

[ref26] Molloy S.; McKeith I. G.; O’Brien J. T.; Burn D. J. The role of levodopa in the management of dementia with Lewy bodies. J. Neurol Neurosurg Psychiatry 2005, 76 (9), 1200–1203. 10.1136/jnnp.2004.052332. 16107351 PMC1739807

[ref27] Nivsarkar M.; Banerjee A. Establishing the probable mechanism of L-DOPA in Alzheimer’s disease management. Acta Polym. Pharm. 2009, 66 (5), 483–486. 19894644

[ref28] Ambree O.; Richter H.; Sachser N.; Lewejohann L.; Dere E.; de Souza Silva M. A.; Herring A.; Keyvani K.; Paulus W.; Schabitz W. R. Levodopa ameliorates learning and memory deficits in a murine model of Alzheimer’s disease. Neurobiol Aging 2009, 30 (8), 1192–1204. 10.1016/j.neurobiolaging.2007.11.010. 18079024

[ref29] Molloy S. A.; Rowan E. N.; O’Brien J. T.; McKeith I. G.; Wesnes K.; Burn D. J. Effect of levodopa on cognitive function in Parkinson’s disease with and without dementia and dementia with Lewy bodies. J. Neurol Neurosurg Psychiatry 2006, 77 (12), 1323–1328. 10.1136/jnnp.2006.098079. 16952917 PMC2077405

[ref30] Dugger B. N.; Serrano G. E.; Sue L. I.; Walker D. G.; Adler C. H.; Shill H. A.; Sabbagh M. N.; Caviness J. N.; Hidalgo J.; Saxon-Labelle M.; Chiarolanza G.; Mariner M.; Henry-Watson J.; Beach T. G.; Presence of Striatal Amyloid Plaques in Parkinson’s Disease Dementia Predicts Concomitant Alzheimer’s Disease: Usefulness for Amyloid Imaging. J. Parkinsons Dis. 2012, 2 (1), 57–65. 10.3233/JPD-2012-11073. 22924088 PMC3423968

[ref31] Nirmalraj P. N.; List J.; Battacharya S.; Howe G.; Xu L.; Thompson D.; Mayer M. Complete aggregation pathway of amyloid beta (1–40) and (1–42) resolved on an atomically clean interface. Sci. Adv. 2020, 6 (15), eaaz6014 10.1126/sciadv.aaz6014. 32285004 PMC7141833

[ref32] Krasnoslobodtsev A. V.; Volkov I. L.; Asiago J. M.; Hindupur J.; Rochet J. C.; Lyubchenko Y. L. alpha-Synuclein misfolding assessed with single molecule AFM force spectroscopy: effect of pathogenic mutations. Biochemistry 2013, 52 (42), 7377–7386. 10.1021/bi401037z. 24066883 PMC3844294

[ref33] Synhaivska O.; Bhattacharya S.; Campioni S.; Thompson D.; Nirmalraj P. N. Single-Particle Resolution of Copper-Associated Annular alpha-Synuclein Oligomers Reveals Potential Therapeutic Targets of Neurodegeneration. ACS Chem. Neurosci. 2022, 13 (9), 1410–1421. 10.1021/acschemneuro.2c00021. 35414168 PMC9073932

[ref34] Bergaglio T.; Bhattacharya S.; Thompson D.; Nirmalraj P. N. Label-Free Digital Holotomography Reveals Ibuprofen-Induced Morphological Changes to Red Blood Cells. ACS Nanoscience Au 2023, 3 (3), 241–255. 10.1021/acsnanoscienceau.3c00004. 37360843 PMC10288613

[ref35] Nirmalraj P. N.; Bhattacharya S.; Thompson D. Accelerated Alzheimer’s Aβ-42 secondary nucleation chronologically visualized on fibril surfaces. Sci. Adv. 2024, 10 (43), eadp5059 10.1126/sciadv.adp5059. 39454002 PMC11506133

[ref36] Gremer L.; Scholzel D.; Schenk C.; Reinartz E.; Labahn J.; Ravelli R. B. G.; Tusche M.; Lopez-Iglesias C.; Hoyer W.; Heise H.; Willbold D.; Schroder G. F. Fibril structure of amyloid-beta(1–42) by cryo-electron microscopy. Science 2017, 358 (6359), 116–119. 10.1126/science.aao2825. 28882996 PMC6080689

[ref37] Walti M. A.; Ravotti F.; Arai H.; Glabe C. G.; Wall J. S.; Bockmann A.; Guntert P.; Meier B. H.; Riek R. Atomic-resolution structure of a disease-relevant Abeta(1–42) amyloid fibril. Proc. Natl. Acad. Sci. U. S. A. 2016, 113 (34), E4976–E4984. 10.1073/pnas.1600749113. 27469165 PMC5003276

[ref38] Best R. B.; Hummer G.; Eaton W. A. Native contacts determine protein folding mechanisms in atomistic simulations. Proc. Natl. Acad. Sci. U. S. A. 2013, 110 (44), 17874–17879. 10.1073/pnas.1311599110. 24128758 PMC3816414

[ref39] Brooks B. R.; Bruccoleri R. E.; Olafson B. D.; States D. J.; Swaminathan S.; Karplus M. CHARMM: A program for macromolecular energy, minimization, and dynamics calculations. J. Comput. Chem. 1983, 4 (2), 187–217. 10.1002/jcc.540040211.

[ref40] Xu L.; Bhattacharya S.; Thompson D. The fold preference and thermodynamic stability of alpha-synuclein fibrils is encoded in the non-amyloid-beta component region. Phys. Chem. Chem. Phys. 2018, 20 (6), 4502–4512. 10.1039/C7CP08321A. 29372732

[ref41] Rochet J. C.; Conway K. A.; Lansbury P. T. Jr. Inhibition of fibrillization and accumulation of prefibrillar oligomers in mixtures of human and mouse alpha-synuclein. Biochemistry 2000, 39 (35), 10619–10626. 10.1021/bi001315u. 10978144

[ref42] Gandhi K. R.; Saadabadi A., Levodopa (L-Dopa). In StatPearls; Treasure Island (FL), 2024.

[ref43] Lee H.-J.; Baek S. M.; Ho D.-H.; Suk J.-E.; Cho E.-D.; Lee S.-J. Dopamine promotes formation and secretion of non-fibrillar alpha-synuclein oligomers. Experimental and Molecular Medicine 2011, 43 (4), 216 10.3858/emm.2011.43.4.026. 21415592 PMC3085740

[ref44] Campioni S.; Carret G.; Jordens S.; Nicoud L.; Mezzenga R.; Riek R. The Presence of an Air–Water Interface Affects Formation and Elongation of α-Synuclein Fibrils. J. Am. Chem. Soc. 2014, 136 (7), 2866–2875. 10.1021/ja412105t. 24460028

[ref45] Nirmalraj P. N.; List J.; Battacharya S.; Howe G.; Xu L.; Thompson D.; Mayer M. Complete aggregation pathway of amyloid β (1–40) and (1–42) resolved on an atomically clean interface. Sci. Adv. 2020, 6 (15), eaaz6014 10.1126/sciadv.aaz6014. 32285004 PMC7141833

[ref46] Lamour G.; Kirkegaard J. B.; Li H.; Knowles T. P. J.; Gsponer J. Easyworm: an open-source software tool to determine the mechanical properties of worm-like chains. Source Code Biol. Med. 2014, 9 (1), 16 10.1186/1751-0473-9-16. 25093038 PMC4106204

