# Supplementary material for: On Levodopa Interactions with Brain Disease Amyloidogenic Proteins at the Nanoscale
Source: ACS Omega. 2025 Apr 2;10(14):14487–95. doi: 10.1021/acsomega.5c01028 (PMC12004170; doi:10.1021/acsomega.5c01028)
Supplement: Supplementary file 1 — ao5c01028_si_001.pdf [file ao5c01028_si_001.pdf]

On Levodopa interactions with brain disease amyloidogenic proteins at the nanoscale

Supporting Information for

Talia Bergaglio[a,b], Nico Kummer[a], Shayon Bhattacharya[c], Damien Thompson[c], Silvia Campioni[d], Peter Niraj Nirmalraj[a,*]

[a]Transport at Nanoscale Interfaces Laboratory, Swiss Federal Laboratories for Materials Science and Technology, Dübendorf CH-8600, Switzerland; [b]Graduate School for Cellular and Biomedical Sciences, University of Bern, Bern CH-3012, Switzerland; [c]Department of Physics, Bernal Institute, University of Limerick, Limerick V94T9PX, Ireland. [d]Functional Materials Laboratory, Swiss Federal Laboratories for Materials Science and Technology, Dübendorf CH-8600, Switzerland

[*]To whom correspondence may be addressed. E-mail: peter.nirmalraj@empa.ch

Preparation of Models and Molecular Dynamics Simulations

The atomically flat gold surface was modelled using a single-atom thick Au(111) slab of surface area 5 nm x 15 nm. By applying periodic boundary conditions (PBC) in the *xy*-plane, the infinite gold surface was modelled, and the Au atoms were held fixed with zero atomic charges. The Aβ-42 fibril structures were modelled as dodecamer (12-mer) in two different folds, one with two symmetric LS-shaped folds of hexamers packed laterally, obtained from the cryo–electron microscopy (cryo-EM) decamer (10-mer) structure of the Aβ-42 fibrils (PDB code 5OQV[1]), and the other with two symmetric double-horseshoe-shaped disease-relevant fold of hexamers packed laterally, and obtained from the solution NMR structure of a hexameric (6-mer) biological unit (PDB code 2NAO[2]). The initial structures of the pre-formed fibrils were oriented with the fibril axis parallel to the gold surface, with the two folds placed laterally on top of gold surface. The two fibril folds were placed at a minimum distance of all atoms of 5 Å above the gold substrate. 1000 molecules of L-Dopa were randomly added inside the simulation cell. To prevent excessive build-up of the L-dopa molecules on gold, the gold atoms were given zero Lennard-Jones parameters and the fibril models were given weak positional constraints on selected alpha carbon atoms to prevent protein dissociation from the gold nanosheet.

The fibril models were represented by the CHARMM36m[3] force field and solvated with CHARMM-modified TIP3P[3] water molecules. The topology and parameter of L-dopa was obtained from CHARMM General Force Field (CGenFF)[4, 5]. Molecular dynamics (MD) simulations were carried out using the Gromacs 2018.4[6, 7] package with a time step of 2 fs

using the leapfrog integrator[8]. Bond lengths to hydrogen in protein were constrained using the LINCS[9] algorithm and water hydrogens were constrained using the SETTLE[10] algorithm. Background counterions were added to neutralise protein formal charges. Long-range electrostatics were treated by the Particle mesh Ewald[11] (PME) method. Protein and non-protein (gold, L-dopa, water and ions) were coupled separately to an external heat bath (298 K) with the coupling time constant of 1 ps using the velocity rescaling[12] method. The four systems (two fibril folds treated with L-dopa and untreated as control) were energy minimised, brought to room temperature over 100 ps and equilibrated for 1 ns in constant volume NVT ensemble followed by another 1 ns of constant pressure NPT equilibration with the reference pressure at 1 bar and a time constant of 4 ps using the Parrinello-Rahman[13, 14] barostat. The production runs were carried out for 480 ns for each of the four systems described above in the NPT ensemble. Structures were saved every 20 ps.

The trajectories were visualized and snapshots captured with Visual Molecular Dynamics (VMD)[15]. All analyses (main text Fig. 5 and Fig. S2 below) of root mean square fluctuation (RMSF), secondary structure and interaction energies analyses were performed using Gromacs tools. The fraction of native contacts was calculated using the definition from Best, Hummer and Eaton[16] implemented in the MDTraj[17] python library. The equation is:

$$Q(X) = \frac{1}{N}\sum_{(i,j)} \frac{1}{1+\exp\left[\beta(r_{ij}(X)-\lambda r_{ij}^{0})\right]} \quad \text{(eqn 1)}$$

where N is the set of all pairs of heavy atoms (i, j), and heavy atoms i and j are in contact if the distance between them is less than 5 Å and are separated by at least 3 residues. rij (X) is the distance between i and j in conformation X and r^0_{ij} is the distance in the native. β is a smoothening parameter taken to be 5 $Å^{-1}$ and the λ is a factor that describes fluctuations when the contact is formed, taken to be 1.8 for the all-atom model. For more details on the methods and parameters, please see ref[16].

The conformational energy was estimated using the GBMV implicit solvent model (Generalized Born using Molecular Volume) implemented in the CHARMM (v40b2) program[18]. The energy was calculated after 200-step minimization of each MD snapshot using the GBMV II algorithm[19-21]. Other energy terms including bonded energy, van der Waals energy, electrostatic energy, and solvation energy were also calculated with the GB implicit solvent model. The block average method was used to estimate the mean values and standard

deviations during the last 100 ns of dynamics, *i.e.*, 1000 statistically independent structures for each system.

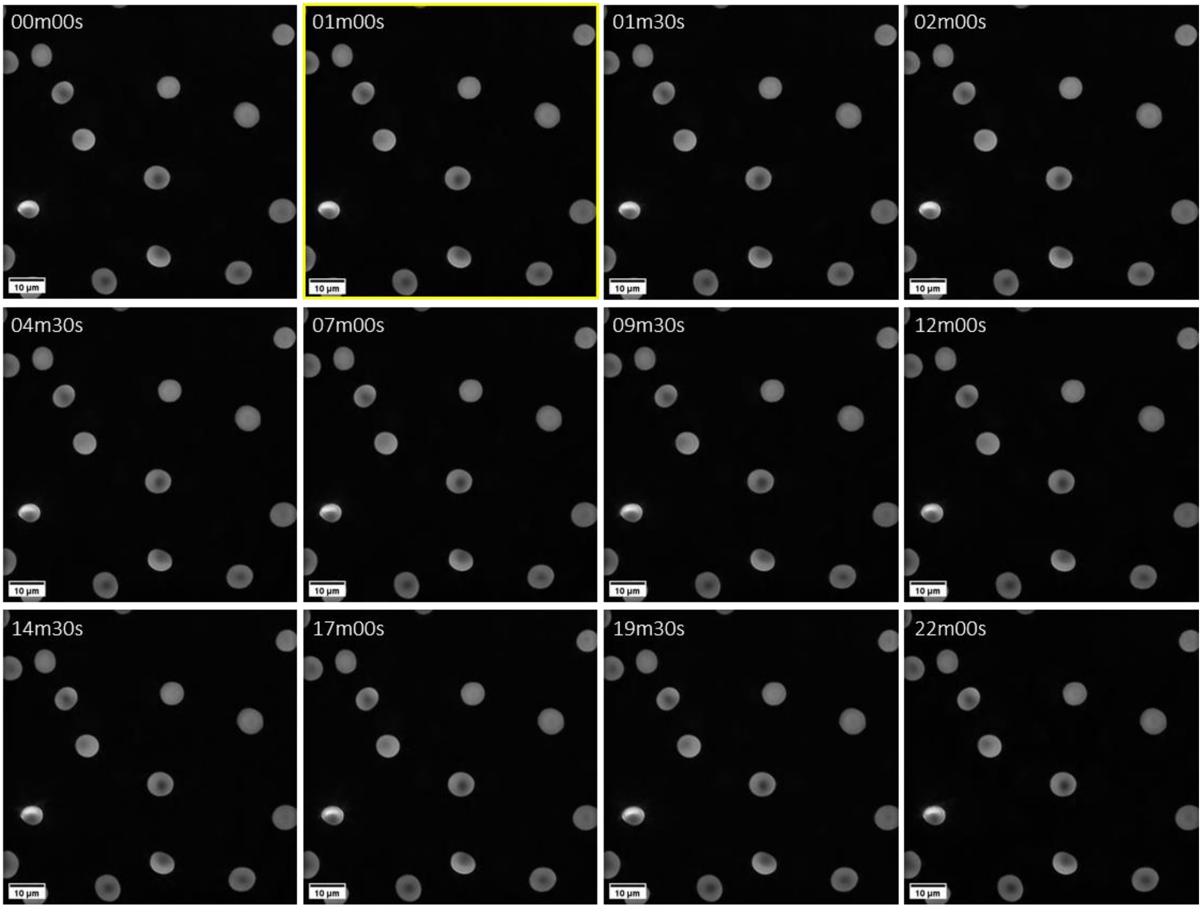

Figure S1:3D holo-tomographic imaging of red blood cells (RBCs) treated with 100 μM L-Dopa solution, during a 22-minute time-lapse. L-Dopa was added after 1 minute (yellow quadrant) from the start of the live cell imaging measurement. No structural alterations to RBC morphology were observed due to L-Dopa treatment.

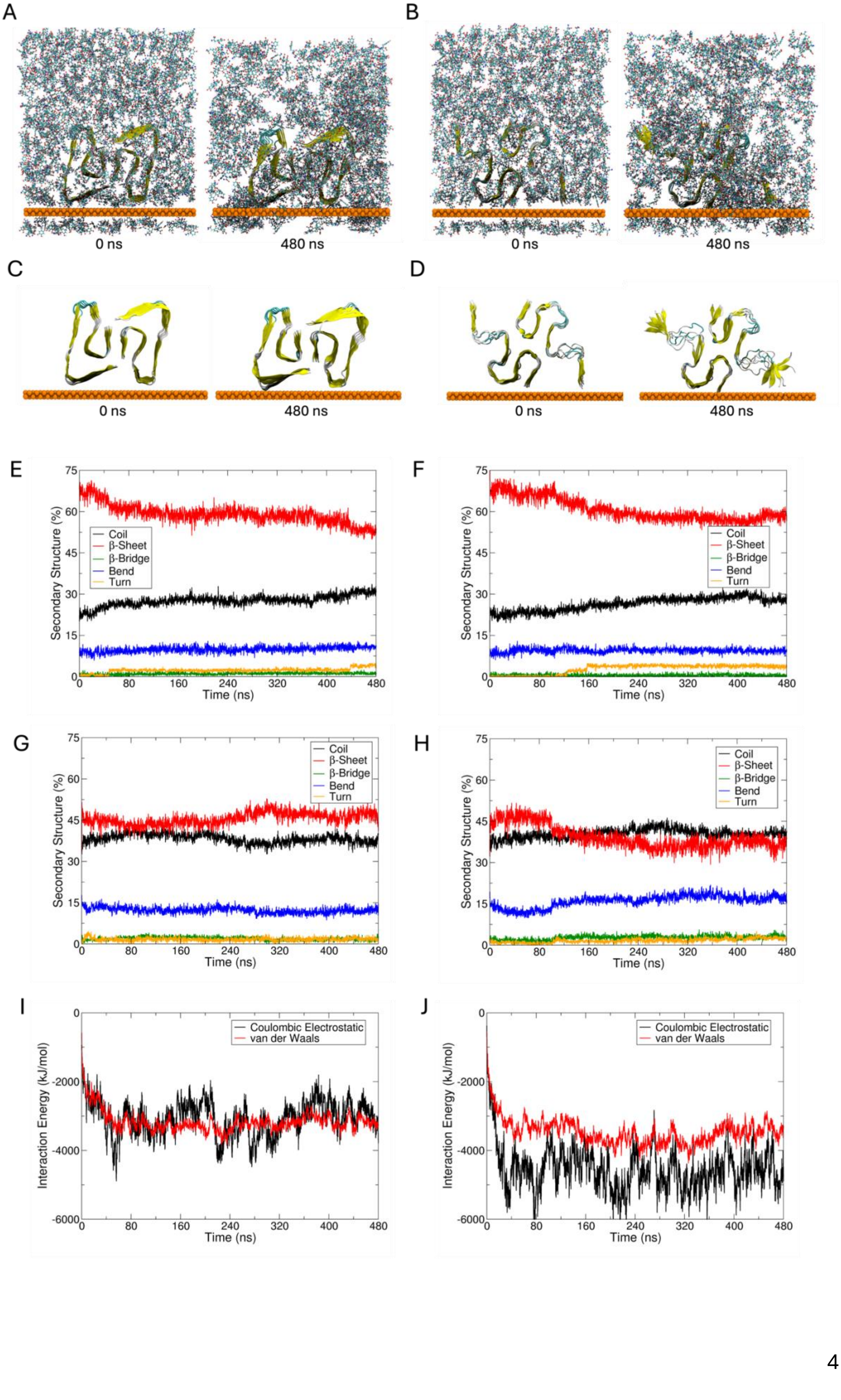
A
0 ns
480 ns
B
0 ns
480 ns
C
0 ns
480 ns
D
0 ns
480 ns
E
F
G
H
Secondary Structure (%)
Time (ns)
Coil
β-Sheet
β-Bridge
Bend
Turn
I
J
Interaction Energy (kJ/mol)
Time (ns)
Coulombic Electrostatic
van der Waals

Figure S2: The initial and final conformations of Aβ-42 fibrils in presence of L-dopa in the **(A)** LS-shaped fold, and **(B)** double-horseshoe-shaped fold from 480 ns MD simulations. The initial and final conformations of Aβ-42 fibrils in absence of L-dopa (control) in the **(C)** LS-shaped fold, and **(D)** double-horseshoe-shaped fold from 100 ns dynamics. Water molecules are omitted for clarity. The time evolution of percentage (%) of secondary structures of **(E)** LS-shaped fibril fold treated with L-dopa, **(F)** untreated LS-shaped fibril fold, **(G)** double horseshoe-shaped fibril fold treated with L-dopa, and **(H)** untreated LS-shaped fibril fold. Decomposition of interaction energies to their Coulombic electrostatic and van der Waals (vdW) components of L-dopa binding to **(I)** LS-shaped and **(J)** double-horseshoe-shaped fibril fold.

Supplementary References

1. Gremer, L.; Scholzel, D.; Schenk, C.; Reinartz, E.; Labahn, J.; Ravelli, R. B. G.; Tusche, M.; Lopez-Iglesias, C.; Hoyer, W.; Heise, H.; Willbold, D.; Schroder, G. F., Fibril structure of amyloid-beta(1-42) by cryo-electron microscopy. *Science* **2017,** *358* (6359), 116-119.
2. Walti, M. A.; Ravotti, F.; Arai, H.; Glabe, C. G.; Wall, J. S.; Bockmann, A.; Guntert, P.; Meier, B. H.; Riek, R., Atomic-resolution structure of a disease-relevant Abeta(1-42) amyloid fibril. *Proc Natl Acad Sci U S A* **2016,** *113* (34), E4976-84.
3. Huang, J.; Rauscher, S.; Nawrocki, G.; Ran, T.; Feig, M.; de Groot, B. L.; Grubmuller, H.; MacKerell, A. D., Jr., CHARMM36m: an improved force field for folded and intrinsically disordered proteins. *Nat Methods* **2017,** *14* (1), 71-73.
4. Vanommeslaeghe, K.; MacKerell, A. D., Jr., Automation of the CHARMM General Force Field (CGenFF) I: bond perception and atom typing. *J Chem Inf Model* **2012,** *52* (12), 3144-54.
5. Vanommeslaeghe, K.; Raman, E. P.; MacKerell, A. D., Jr., Automation of the CHARMM General Force Field (CGenFF) II: assignment of bonded parameters and partial atomic charges. *J Chem Inf Model* **2012,** *52* (12), 3155-68.
6. Abraham, M. J.; Murtola, T.; Schulz, R.; Páll, S.; Smith, J. C.; Hess, B.; Lindahl, E., GROMACS: High performance molecular simulations through multi-level parallelism from laptops to supercomputers. *SoftwareX* **2015,** *1*, 19-25.
7. Van Der Spoel, D.; Lindahl, E.; Hess, B.; Groenhof, G.; Mark, A. E.; Berendsen, H. J., GROMACS: fast, flexible, and free. *J Comput Chem* **2005,** *26* (16), 1701-18.
8. Cuendet, M. A.; van Gunsteren, W. F., On the calculation of velocity-dependent properties in molecular dynamics simulations using the leapfrog integration algorithm. *The Journal of chemical physics* **2007,** *127* (18), 184102.
9. Hess, B.; Bekker, H.; Berendsen, H. J.; Fraaije, J. G., LINCS: a linear constraint solver for molecular simulations. *Journal of computational chemistry* **1997,** *18* (12), 1463-1472.
10. Miyamoto, S.; Kollman, P. A., Settle: An analytical version of the SHAKE and RATTLE algorithm for rigid water models. *Journal of computational chemistry* **1992,** *13* (8), 952-962.
11. Darden, T.; York, D.; Pedersen, L., Particle mesh Ewald: An N· log (N) method for Ewald sums in large systems. *The Journal of chemical physics* **1993,** *98* (12), 10089-10092.

12. Bussi, G.; Donadio, D.; Parrinello, M., Canonical sampling through velocity rescaling. *J Chem Phys* **2007,** *126* (1), 014101.
13. Parrinello, M.; Rahman, A., Polymorphic transitions in single crystals: A new molecular dynamics method. *Journal of Applied physics* **1981,** *52* (12), 7182-7190.
14. Parrinello, M.; Rahman, A., Crystal structure and pair potentials: A molecular-dynamics study. *Physical Review Letters* **1980,** *45* (14), 1196.
15. Humphrey, W.; Dalke, A.; Schulten, K., VMD: visual molecular dynamics. *J Mol Graph* **1996,** *14* (1), 33-38.
16. Best, R. B.; Hummer, G.; Eaton, W. A., Native contacts determine protein folding mechanisms in atomistic simulations. *Proc Natl Acad Sci U S A* **2013,** *110* (44), 17874-9.
17. McGibbon, R. T.; Beauchamp, K. A.; Harrigan, M. P.; Klein, C.; Swails, J. M.; Hernandez, C. X.; Schwantes, C. R.; Wang, L. P.; Lane, T. J.; Pande, V. S., MDTraj: A Modern Open Library for the Analysis of Molecular Dynamics Trajectories. *Biophys J* **2015,** *109* (8), 1528-32.
18. Brooks, B. R.; Bruccoleri, R. E.; Olafson, B. D.; States, D. J.; Swaminathan, S.; Karplus, M., CHARMM: A program for macromolecular energy, minimization, and dynamics calculations. *Journal of Computational Chemistry* **1983,** *4* (2), 187-217.
19. Lee, M. S.; Salsbury, F. R.; Brooks, C. L., Novel generalized Born methods. *The Journal of Chemical Physics* **2002,** *116* (24), 10606-10614.
20. Lee, M. S.; Feig, M.; Salsbury, F. R.; Brooks, C. L., New analytic approximation to the standard molecular volume definition and its application to generalized Born calculations. *Journal of Computational Chemistry* **2003,** *24* (11), 1348-1356.
21. Feig, M.; Onufriev, A.; Lee, M. S.; Im, W.; Case, D. A.; Brooks, C. L., Performance comparison of generalized born and Poisson methods in the calculation of electrostatic solvation energies for protein structures. *Journal of Computational Chemistry* **2004,** *25* (2), 265-284.
